# OAM-inspired new optics: the angular metalens

**DOI:** 10.1038/s41377-021-00541-6

**Published:** 2021-05-06

**Authors:** Gianluca Ruffato

**Affiliations:** Department of Physics and Astronomy ‘G. Galilei’, University of Padova, Padova, Italy

**Keywords:** Optics and photonics, Applied optics

## Abstract

Analogous to the behavior of a common converging lens for the input of tilted waves, a recent publication suggests a new optical element with an azimuthal-quadratic phase profile for the focusing of orbital angular momentum beams at distinct angular positions. Its realization in a metasurface form enables the combined measurement of orbital and spin angular momentum using a single optical component.

The demand for the control and exploitation of light has always boosted the progress of human technology. Basically, the history of optics coincides with the improving ability to manipulate matter to realize new optical elements with better quality and advancing performance. Since G. Galilei pointed his telescope toward the sky, reporting the first detailed observations of the Moon and satellites of Jupiter in 1609-10^1^, lenses have become the essential components to build scientific instruments that extend the human gaze out to the stars and down to the microscale. Their refinement has provided a wide range of optical instruments and applications in astronomy, microscopy, and communications. The need to exploit additional degrees of freedom, such as wavelength and polarization, has led to the introduction of new optical elements, but it is ultimately space that has ignited the development of many innovative devices and techniques to tailor the intensity and phase structure of light beams^2^.

Beams carrying orbital angular momentum (OAM) have gained a preeminent role^3^, paving the way for scientific milestones and innovative applications in an amazing variety of fields^4,5^, such as micromanipulation, microscopy and imaging, laser material processing, and classical and quantum communications. In particular, in information and communication technology, OAM has probably opened some of the most promising fields of application, offering a wider state space for enhanced-capacity transmission^6^ and high-alphabet quantum protocols^7^.

The need to generate and measure OAM has promoted the development of many methods and techniques with different levels of complexity and performance. Among all, lossless and scalable OAM decomposition is based on two-element conformal mappings^8,9^ or multiplane light conversion^10^. While polarization or frequency analysis can be done straightforwardly, spatial decomposition is inherently more arduous; hence, very few practical examples exist. For instance, a simple lens is capable of performing only a spatial decomposition over the trivial basis of plane waves, i.e., linear momentum states. The input field is transformed into a bright spot on the focal plane, with the axial displacement depending on the linear phase gradient (Fig. 1a).

**Fig. 1:**
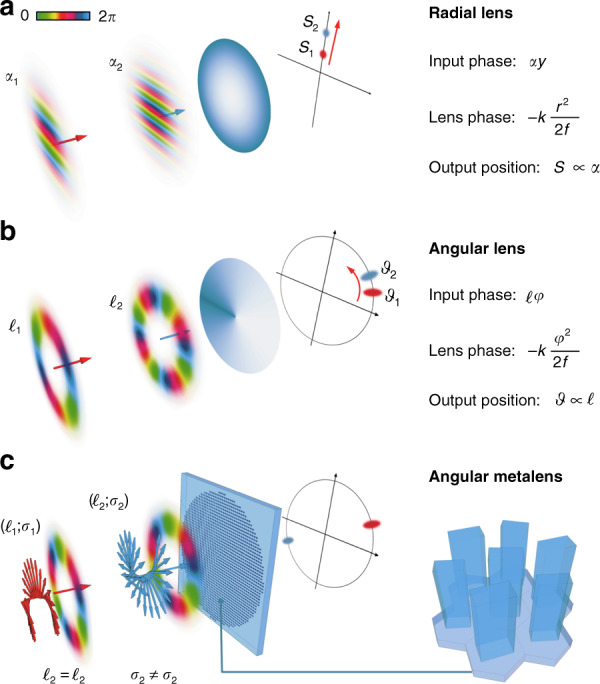
Working principle of the angular lens. **a** A radial-quadratic phase focuses different linear phase gradients onto distinct axial positions. **b** An azimuthal-quadratic phase performs an analogous operation on orbital angular momentum (OAM) beams, mapping different azimuthal phase gradients onto distinct angular positions. **c** Its implementation in a metasurface form enables the combined measurement of polarization (spin *σ*) and OAM

OAM beams are endowed with azimuthal phase gradients, which can be seen as linear phase gradients wrapping around the propagation direction. Analogous to the abovementioned effect of a radial-quadratic phase, approximating a standard lens, on linear phase gradients, one can imagine applying an angular-quadratic phase to the azimuthal gradient of an OAM beam (Fig. 1b). Introduced for the first time in 2018^11^ under the name of an “angular lens” and tested with spatial light modulators, this new optical element was lately rediscovered and investigated in a very recent publication in the journal *Light: Science & Applications*^12^. In this paper, the authors present a single azimuthal-quadratic phase mask that generates V-shaped cusp caustics with angular positions that are proportional to the value of the input OAM-beam illumination.

The optical element is implemented in a metasurface form by designing a pattern of dielectric (TiO_2_) rectangular nanopillars with spatially variant geometries distributed over a hexagonal lattice. By controlling both the local cross-section and orientation of the TiO_2_ metaunits, the resulting effective medium allows one to simultaneously act on the dynamic and geometric phases, respectively. In this way, it is possible to engineer a spin-controlled dual-functional metasurface that is able to optically process the two orthogonal circularly polarized states in different ways. In this specific case, the technique is used to focus beams with the same OAM and orthogonal circular polarizations at distinct (opposite) angular positions, enabling the measurement of both the orbital and spin angular momentum with a single optical component (Fig. 1c). The analysis of cylindrical vector beams confirms the capability of the optics to sort the constituent circularly polarized OAM contributions, while the geometric nature of the phase allows exploitation within a broad visible band.

Combining the dynamic phase from the refractive index profile and the geometric phase from the induced spatially variant form-birefringence implies that light can be manipulated in a more sophisticated way than usual. Metasurfaces represent the ultimate evolution of optics, providing a new generation of flat (digital) components with an increased density of features and functionalities^13^. While the underlying concepts of effective-medium engineering date back to metamaterial design, it has been the pioneering work of F. Capasso’s group to show all the potentialities of the metasurface paradigm for the realization of multifunctional dielectric metalenses^14^. This paradigm shift rapidly encompasses the field of structured light with the implementation of digital high-index metaoptics and tunable liquid-crystal devices for OAM-beam generation^15^ and conformal sorters^16–19^.

The article by Yinghui Guo et al. confirms this trend toward integration and compactness and suggests a new solution for a total angular momentum measurement that employs a single optical element. While the present efficiency values make this technique not ideal for single-photon applications, it deserves further study for many-photon applications where a compact and combined measurement of spin and OAM is required. We envisage that further engineering of the phase profile can optimize the caustic pattern, thereby improving the OAM-detection resolution and overall efficiency. Furthermore, the choice of a more complex metaunit design can open additional control of the group delay and group delay dispersion to achieve full achromaticity. Inspired by the azimuthal phase of OAM beams, the angular lens further extends the portfolio of methods to process complex light fields and proves how the evolution of optical components and their design techniques is still a dynamic and growing process.
